# Immunoexpression of aromatase cytochrome P450 and 17β-hydroxysteroid dehydrogenase in women’s ovaries after menopause

**DOI:** 10.1186/1757-2215-7-52

**Published:** 2014-05-10

**Authors:** Agnieszka Brodowska, Jacek Brodowski, Maria Laszczyńska, Sylwia Słuczanowska-Głąbowska, Bogdan Rumianowski, Iwona Rotter, Andrzej Starczewski, Mariusz Z Ratajczak

**Affiliations:** 1 Department of Gynaecology and Urogynaecology, Pomeranian Medical University, Siedlecka 2, 72 – 010 Police, Poland; 2 Laboratory of Primary Health Care, Pomeranian Medical University, Żołnierska 48, 71-210 Szczecin, Poland; 3 Department of Histology and Developmental Biology, Pomeranian Medical University, Żołnierska 48, 71-210 Szczecin, Poland; 4 Department of Physiology Pomeranian Medical University, Powstańców Wielkopolskich 72, 70-111 Szczecin, Poland; 5 Laboratory of Medical Rehabilitation, Pomeranian Medical University, Grudziądzka 31, 70-103 Szczecin, Poland; 6 Stem Cell Biology Program, James Graham Brown Cancer Center, University of Louisville, Louisville, KY, USA

**Keywords:** Women’s ovaries, Menopause, Steroidogenesis, Aromatase cytochrome P450, 17β-hydroxysteroid dehydrogenase, Immunexpression

## Abstract

**Background:**

Menopause results in a lack of regular menstrual cycles, leading to the reduction of estrogen production. On the other hand, ovarian androgen synthesis is still present at reduced levels and requires expression of several steroidogenic enzymes.

**Methods:**

This study was performed on 104 postmenopausal women hospitalized due to uterine leiomyomas, endometriosis, and/or a prolapsed uterus. Patients were divided into three groups depending on the time from menopause. Group A patients experienced menopause 1–5 years before enrollment in the study (42 women). Group B included women who had their last menstruation 5–10 years before the study (40 women). Group C consisted of 22 women who were more than 10 years past menopause. Hysterectomy or removal of the uterine corpus with adnexa was performed during laparotomy. We evaluated the expression of aromatase cytochrome P450 (CYP 19) and 17β-hydroxysteroid dehydrogenase (17β HSD) by employing immunohistochemistry.

**Results:**

Activity of 17β-HSD and CYP19 was demonstrated in the cytoplasm of stromal cells of postmenopausal ovaries, epithelium cells coating the ovaries, vascular endothelial cells, and epithelial inclusion cysts. However, overall expression of both 17β-HSD and CYP 19 decreased with time after menopause.

**Conclusion:**

Demonstration of the activity of the key enzymes of ovarian steroidogenesis, CYP 19 and 17β-HSD, confirms steroidogenic activity in the ovaries of postmenopausal women. Nevertheless, ovarian steroidogenic activity decreases with time, and its significant decrease occurs 10 years after menopause.

## Introduction

The key enzymes of ovarian steroidogenesis are hydroxylases and oxidases, which belong to a large family of cytochrome P450 [1-4] that consists of 480 enzymes, including i) P450scc, which is responsible for cleaving the side chain of cholesterol, ii) P450c11, which mediates the conversion of 11-deoxycorticosterone to corticosterone and possesses 11-hydroxylase, 18-hydroxylase, and 19-methylooxydase activities, iii) P450c17, which mediates 17-hydroxylation of pregnenolone and progesterone, iv) P450c21, which has 21-hydroxylase activity, and v) aromatase cytochrome P450, whose activity directs aromatisation of androgens to estrogens [4-8]. Steroidogenesis includes a few characteristic reactions such as i) side-chain cleavage as a result of desmolase activity, ii) conversion of hydroxyl groups to ketone groups mediated by dehydrogenase, and iii) addition of hydroxyl groups (hydroxylation) by a reaction involving the formation of double bonds (removing hydrogen atoms) and saturation (adding hydrogen atoms) [3,4].

The most important step in women’s steroidogenesis is aromatisation of androgens, which leads to the generation of estrogens. This process is mediated by aromatase cytochrome P450 and is observed in the endoplasmic reticulum of granulosa cells in many steroidogenic and non-steroidogenic tissues and glands, including the ovaries, placenta, adipose tissue, hair, vascular smooth muscle, skin, liver, bones (osteoblasts), and blood vessels [4-6,8,9].

The human gene encoding aromatase cytochrome P450 is located on the long arm of chromosome 15, region 21.2 (15q21.2) and is called CYP19. This name reflects enzymatic oxidation of the methyl group at C-19 [3,4,10]. In the ovary, aromatase cytochrome P450 catalyzes conversion of testosterone (T) to estradiol (E_2_), androstendione (A) to estrone (E_1_), and 16α-hydroxylated dehydroepiandrosterone (DHEA) to estriol (E_3_) [5,6,11]. In women of reproductive age, 70% of the E_1_ and over 95% of the E_2_ circulating in serum are ovary-derived [4,8]. The rest of the serum-circulating estrogens come from peripheral conversion of androgens, mainly due to conversion of androstendione [5-7,11].

Unlike most of the enzymes involved in steroidogenesis, CYP 19 is expressed in target tissues with active steroidogenesis (ovaries, placenta) as well as in other non-steroidogenic tissues [11-13]. A number of studies have confirmed a key role of CYP 19 in steroidogenesis, especially in aromatisation of androgens to estrogens. Patients lacking this complex suffer from hypergonadotropic hypogonadism [14-16]. On the other hand, aromatase inhibitors are the most suitable drugs for inhibiting estrogen biosynthesis in some estrogen-sensitive diseases, such as breast cancer, ovulation disorders, endometriosis, fibroids, or precocious puberty [17-23].

**Figure 1:**
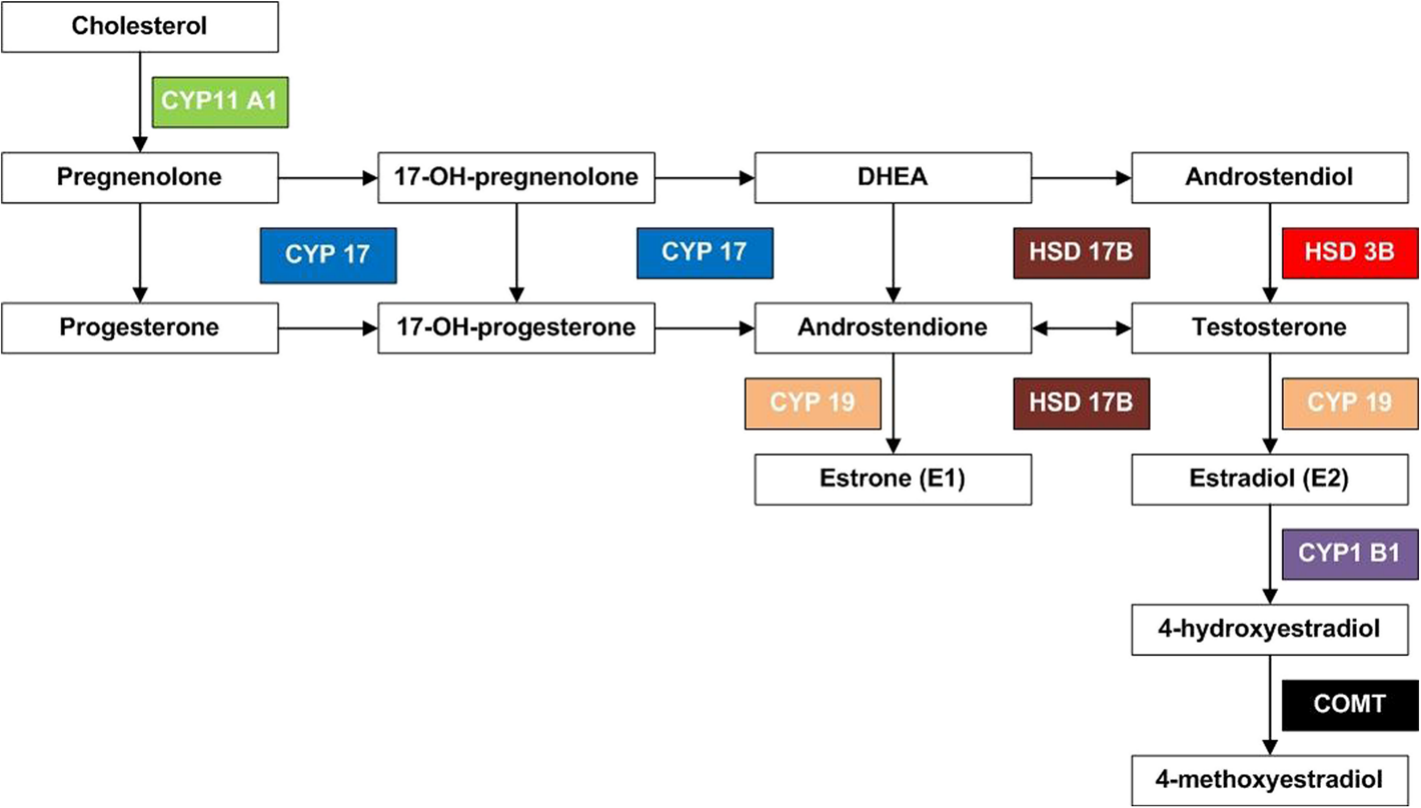
Scheme of ovarian steroidogenesis.

The regulation of estrogen activity depends on oxidation and reduction reactions mediated by isoforms of 17β-hydroxysteroid dehydrogenase (17β-HSD), which catalyze the conversion of less-active 17-keto steroids to more potent 17β-hydroxy steroids, and many isoforms of this enzyme have been identified, differing in expression profiles, regulatory mechanisms, and substrates. The scheme of ovarian steroidogenesis is presented in Figure 1[1,2,7,8,24-30].

Due to the recently widely discussed existence of postmenopausal ovarian steroidogenic activity and the influence of the ovaries on women’s postmenopausal life quality and overall life span we evaluated the immunoexpression of two key ovarian enzymes regulating steroidogenesis: CYP 19 and 17β-HSD in the ovaries of postmenopausal women. These observations are also essential for pre- and postmenopausal women with benign ovarian tumors to facilitate decision if the oophorectomy is necessary.

This study extends our previous work on the morphological structure of the postmenopausal ovary [31,32] and immunoexpression of the androgen, estrogen, and progesterone receptors [33,34] and the follicle stimulating and luteinizing hormone receptors [35].

## Methods

### Study group

The study group consisted of 104 postmenopausal women hospitalized due to uterine leiomyomas, endometriosis, and/or a prolapsed uterus between 2003 and 2008 at the Department of Reproduction and Urogynecology at the Pomeranian Medical University in Szczecin. All women subjects met the inclusion criteria and didn’t meet the exclusion criteria. The inclusion criteria were: i) 12 or more months since the last menstruation, ii) no postmenopausal hormonal therapy, iii) normal PAP smear and mammography, iv) normal blood pressure, v) no history of surgeries that could affect blood flow to the ovaries, and vi) no history of cancer or endocrine disorders such as thyroid disease or diabetes. The research plan was approved by the Bioethics Commission of the Pomeranian Medical University, and the research was supported by grant No. 2 PO5E-10527 from the Polish State Committee for Scientific Research.

Patients who met the inclusion criteria were divided into three groups (A, B, and C), depending on the time from menopause. Group A patients experienced menopause 1–5 years before being qualified for the study (42 women). Group B included women who had their last menstruation 5–10 years before the study (40 women). Group C consisted of 22 women who were at least 10 years post menopause. For ethical reasons, a control group was not studied.

### Immunohistochemistry (IHC)

Hysterectomy or removal of the uterine corpus with adnexa was performed during laparotomy and the “wedge shape” samples of ovaries were obtained for histological analysis. To determine the expression of aromatase cytochrome P450 and 17β-HSD by IHC, samples were preserved in 4% buffered formalin solution and embedded in paraffin. Histological sections were placed in the pH 9.0 buffer and then boiled for 30 minutes in a water bath at 99°C. All sections were then incubated for 24 hours at 4°C in a humid chamber with MCA 2077S primary antibody (Serotec, USA) to detect CYP 19 and with HPA015307 primary antibody (Sigma, USA) to detect 17β-HSD.

For anti-aromatase antibody detection, the ABC Staining System (Santa Cruz Biotechnology, USA) was used, while for anti-17β-HSD primary antibody detection, labeled polymer from the Dako Real EnVision Detection System (Dako, Denmark) was used. In both cases, to visualize the immunohistochemical reaction, 3,3’diaminobenzidine (DAB) was used. In the last step, sections were counterstained with Meyer’s hematoxylin. For the negative control, specific antibodies were omitted in the staining procedure. The slides were examined by employing light microscope (Olympus BX41).

## Results

**Figure 2:**
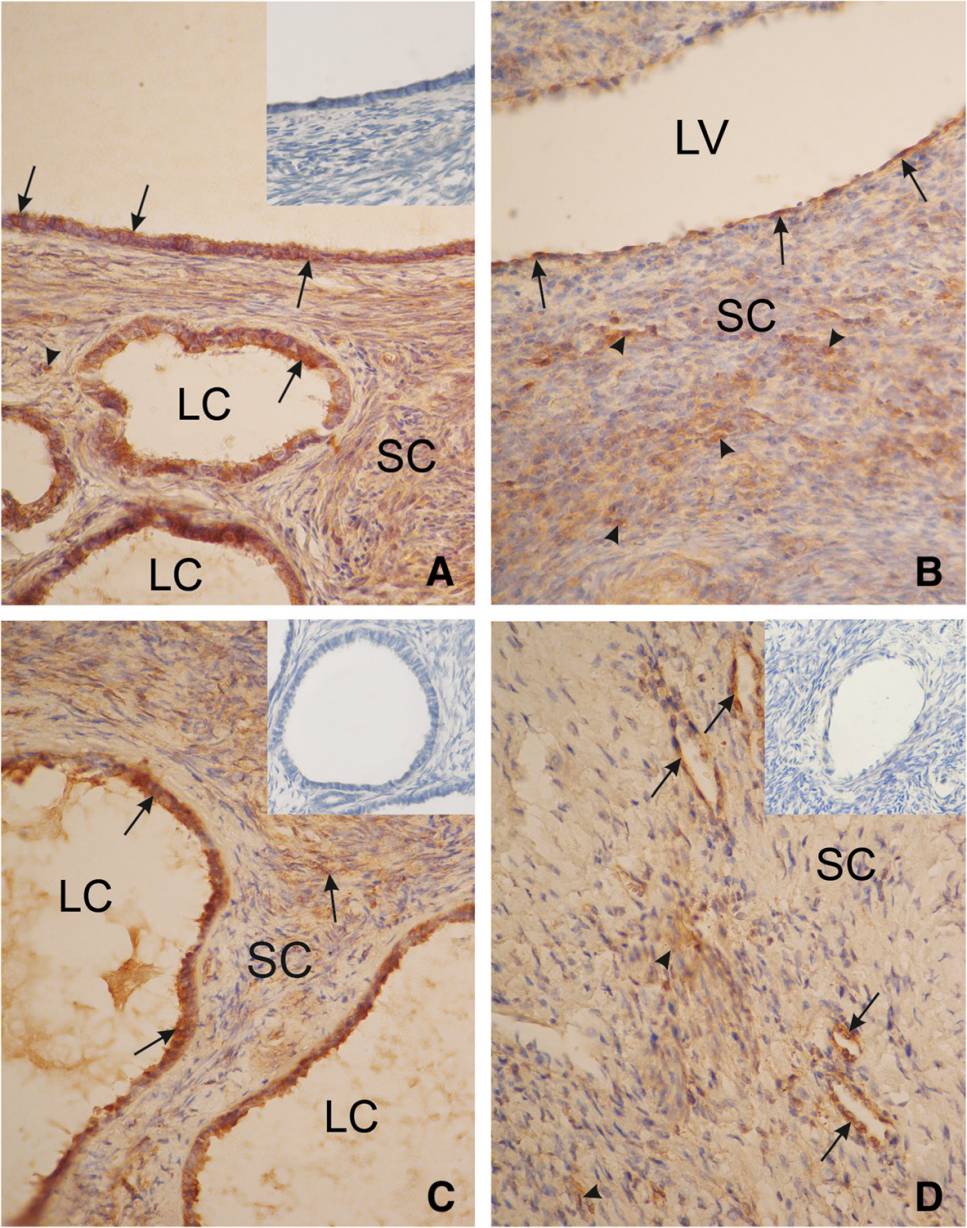
**An ovary of a patient from group A (<5 years after menopause).** Strong cytoplasmic reaction of 17β-HSD in the surface epithelium (arrow), in the cyst epithelium (arrow) **(A)**, in the ovarian stromal cells (arrowhead), and semi strong reaction in the vascular endothelial cells (arrow) **(B)**. An ovary from a patient from group B (5–10 years after menopause). Strong cytoplasmicreaction of 17β-HSD in the cyst epithelium (arrow) and semi strong in ovarian stromal cells (arrowhead) **(C)**. An ovary of a patient from group C (>10 years after menopause). Semi strong cytoplasmic expression of 17β- HSD in the vascular endothelial cells (arrow) and weak expression in the ovarian stromal cells **(D)**. SC, stromal cells; LC, lumen cyst; LV, lumen vessel. Inserts in panels **A**, **C** and **D**) show negative controls. Magnification, x330.

**Figure 3:**
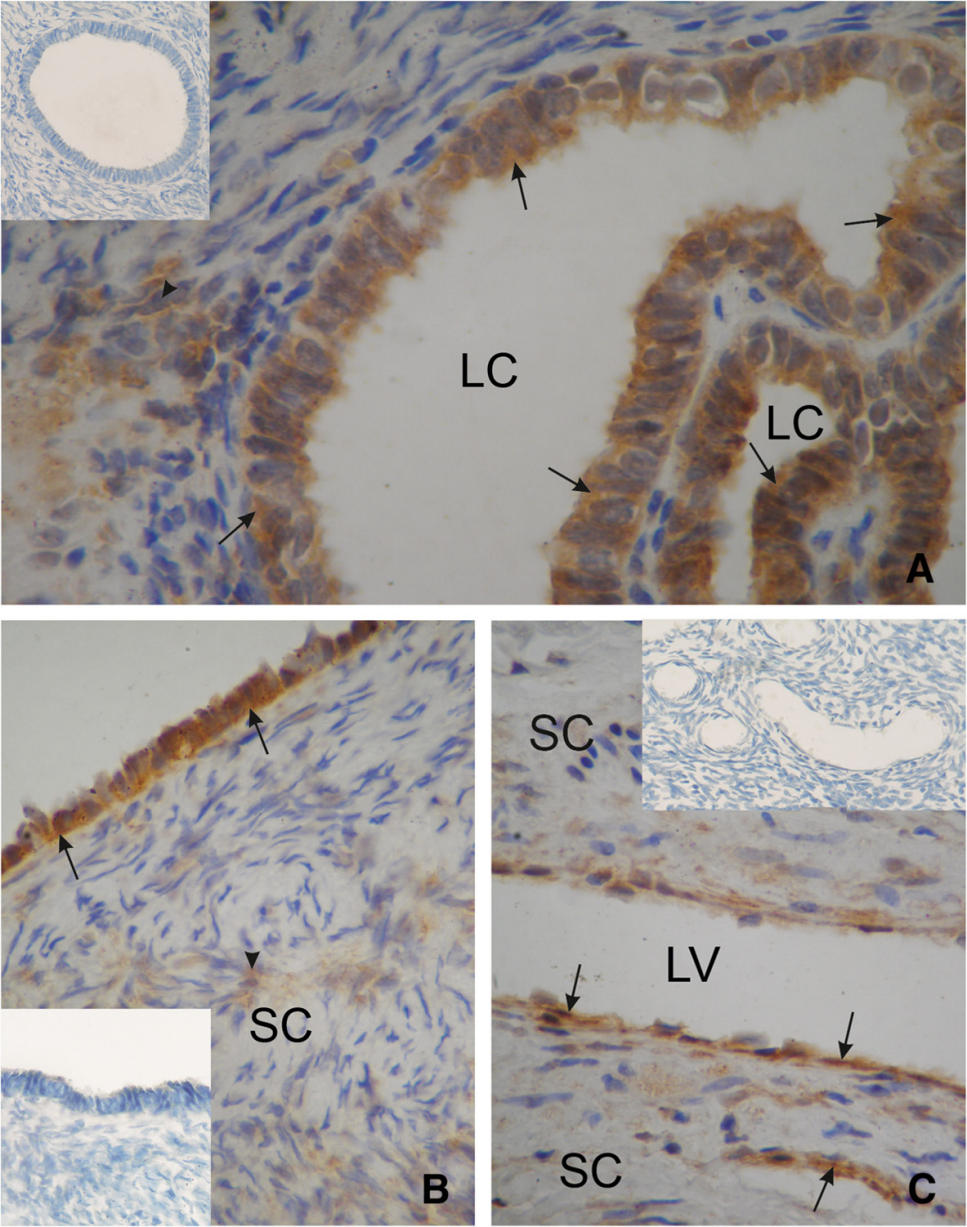
**An ovary of a patient from group A (<5 years after menopause).** Strong cytoplasmic reaction of CYP 19 the cyst epithelium (arrow), and semi strong in the ovarian stromal cells (arrowhead) **(A)**. An ovary of a patient from group B (5–10 years after menopause). Strong cytoplasmic reaction of CYP 19 in the surface epithelium (arrow) and weak in ovarian stromal cells (arrowhead) **(B)**. An ovary of a patient from group C (>10 years after menopause). Cytoplasmic expression of CYP 19 in the vascular endothelial cells (arrow) **(C)**. SC, stromal cells; LC, lumen cyst; LV, lumen vessel. Inserts in panels A, B and C show negative controls. Magnification, x 660.

17β-HSD (Figure 2) and CYP 19 (Figure 3) activity were detected in the cytoplasm of stromal cells of postmenopausal ovaries, epithelium cells coating the ovaries, vascular endothelial cells, and in epithelial inclusion cysts.

In group A, a strong intensity of both activities was demonstrated in epithelial inclusion cysts, epithelial cells coating the ovaries, and in the cytoplasm of stromal cells. The intensity of both reactions was strong and/or semi strong in vascular endothelial cells.

In group B, strong immunoexpression of 17β-HSD was demonstrated in epithelial inclusion cysts and semi strong in ovarian stromal cells. Semi strong reaction was also observed in vascular endothelial cells, and in epithelium cells of the ovary. In the same group, semi strong or weak immunoexpression of CYP 19, was demonstrated in ovarian stromal cells, a semi strong reaction was observed in vascular endothelial cells, and epithelial inclusion cysts, while in and epithelium cells coating the ovary, the reaction was strong.

In group C, the immunoexpression of 17β-HSD and CYP 19, in all studied ovarian structures (i.e., stromal cells of the postmenopausal ovaries, epithelium cells coating the ovaries, vascular endothelial cells and epithelial inclusion cysts) was weak or weak and semi strong in vascular endothelial cells.

We observed that immunoexpression of both 17β-HSD and CYP 19 decreased with time after menopause (Tables 1 and 2).

**Table 1:** Immunoexpression of 17β-HSD in the ovaries of postmenopausal women, depending on the time after menopause

**Study group**	**Epithelium coating the ovaries**	**Epithelial inclusion cysts**	**Vascular endothelial cells**	**Stromal cells**
Group A	+++	+++	+++/++	+++
(n = 42)				
Group B	++	+++	++	++
(n = 40)				
Group C	+	+	++/+	+
(n = 22)				

(+++) strong reaction, (++) semi strong reaction, (+) weak reaction.

**Table 2:** Immunoexpression of aromatase cytochrome P450 in the ovaries of postmenopausal women, depending on the time from menopause

**Study group**	**Epithelium coating the ovaries**	**Epithelial inclusion cysts**	**Vascular endothelial cells**	**Stromal cells**
Group A	+++	+++	+++/++	+++/++
(n = 42)				
Group B	+++	++	++	++/+
(n = 40)				
Group C	+	+	++/+	+
(n = 22)				

(+++) strong reaction, (++) semi strong reaction, (+) weak reaction.

## Discussion

The results presented here are the first concerning the immunoexpression of CYP 19 and17β-HSD in the human ovary of postmenopausal women. Our studies demonstrated CYP 19 and 17β-HSD expression in the ovarian surface epithelial cells, inclusion cysts, vascular endothelial cells, and stromal cells. The intensity of reaction decreased significantly with time after the last menstruation. The activity of key enzymes involved in gonadal steroidogenesis, such as 17β-HSD and CYP 19, provides strong evidence for the presence of the steroid biosynthesis in the postmenopausal ovaries. These results also indicate a reduction in the activity of gonadal steroidogenesis as a function of time elapsed from menopause. Thus, our results confirm the presence of relatively high concentrations of steroids in ovarian tissue homogenates and their reduction in postmenopausal women, especially in women 10 years after the last menstruation [8,12].

The activity of key ovarian steroidogenesis enzymes in postmenopausal women are estimated with different methods, because the ovarian stromal cells represent a heterogeneous collection of cells [36-38].

The presence of enzymes of the aromatase cytochrome group was detected employing immunohistochemistry in the ovaries of postmenopausal women for the first time by Inkster and Brodie in 1991 [19]. On the other hand, the activities of different types of 17β-HSD have been found in many of the human tissues examined, the highest estrogenic activity of 17β- HSD was found in ovary, endometrium, prostate, testis, placenta, liver, and adipose tissue [19]. In 2006 Havelock *et al.* showed that all enzymes necessary for androgen synthesis are present in the ovaries of postmenopausal women [19]. In their study, using the QRT method, the presence of CYP 19 and 17β-HSD and decreased activity of 3β-HSD were detected. Additionally, as reported for the ovaries of postmenopausal women, the delta-5 pathway of steroidogenesis is favored, which leads to synthesis DHEA and its sulfate (DHEAS), which are peripherally converted to testosterone, dihydrotestosterone, androstendione, and estrogens [19]. However, in this latter study, no tissue localization of enzymes involved in this process has been demonstrated.

Our current study demonstrates localization of two key enzymes involved in the steroidogenic biosynthesis pathway, CYP 19 and 17β-HSD, in postmenopausal ovaries, which indicates that the postmenopausal ovary acts as an important contributor to the synthesis of steroids destined for peripheral metabolism.

Studies on the pathogenesis of diseases of the perimenopausal period identified aromatase activity not only in gonadal tissues but also in adipose tissue, muscles, mammary gland, and liver [39-41]. Moreover, the activity of aromatase located in tissues other than gonad is not FSH-dependent [4,8,39,41-44]. Therefore, the application of aromatase inhibitors in women results in a significant reduction in estrogen synthesis [39,41], as seen, for example, in women with breast cancer, where the conversion of androgens to estrogens takes place directly in the tumor cells [39,45,46].

Different types of 17β-HSD use various substrates. The most active type, 17β-HSD1, is highly complementary to estrogenic substrates, especially E_1_, which is converted in the gonads and many peripheral tissues to E_2_. In transgenic mice, increased activity of 17β-HSD1 in the presence of E_1_ substrate leads to increased estrogen-dependent tumor growth. This was confirmed by administration of 17β-HSD1 inhibitor, which significantly reduced growth of this estrogen-dependent tumor [26]. The presence of 17β-HSD in the postmenopausal ovary suggests steroidogenic activity in this tissue. As is well known, the ovaries are not the only source of estrogens in women. 17β-HSD is also involved in peripheral E_2_ formation, despite the fact that its expression in peripheral tissues is low [47].

A few studies demonstrated that genetic polymorphism of the steroidogenic enzymes in postmenopausal women’s ovaries cause dysfunction and may be a cause of increased risk of endometrial and breast cancers. Postmenopausal women with specific CYP19 genetic polymorphisms are at increased risk of breast cancer, and there were higher CYP19 and 17β-HSD activities in tumor cells than in normal cells. Moreover, CYP 19 activity was higher in women with breast and endometrial cancers than in healthy women [48-50], and 17β-HSD activity was higher in breast cancer patients [51-53]. Undoubtedly, research on key gonadal and extragonadal steroidogenesis enzymes and their genetic polymorphisms should be continued, because given the nature of the postmenopausal period, it is very difficult to demonstrate a positive impact of the gonads on life quality, survival, and cancer risk in women of that age.

On the one hand, it is known that postmenopausal women with preserved gonads have longer survival, higher life quality, and less-frequent cardiovascular complications, dementing illnesses, and osteoporotic complications. On the other hand, the presence of gonads increases the risk of ovarian and breast cancer. The U.S. data show that the proportion of deaths from cardiovascular diseases in postmenopausal women is much higher than from cancer, which is confirmed by data from the Central Statistical Office in Poland for the years 2009–2010. Our own data and data from the published literature also indicate that gonadal steroidogenic activity clearly decreases after 10 years from menopause, and in this period, preventive bilateral ovariectomy does not affect life quality and survival time but significantly reduces the risk of cancer.

## Conclusions

Our data support the conclusion that steroidogenesis, as evidenced by activities of the key ovarian steroidogenic enzymes, CYP 19 and 17β-HSD, is still active in the ovaries of postmenopausal women. This process decreases gradually after menopause and is significantly reduced after 10 years from the last menstruation. Therefore taking into consideration hormonal activity in postmenopausal ovaries, prophylactic, bilateral ovariectomy can be safely recommended in the first 10 years after the last menstruation.

## Abbreviations

17βHSD: 17β-hydroxysteroid dehydrogenase; 17βHSD1: 17β-hydroxysteroid dehydrogenase type 1; 3βHSD: 3β-hydroxysteroid dehydrogenase; A: Androstendione; CYP19: Aromatase cytochrome P450; DAB: Diaminobenzidine; DHEA: Dehydroepiandrosterone; DHEAS: Dehydroepiandrosterone sulfate; E2: Estradiol; E3: Estriol; E1: Estrone; FSH: Follicle-stimulating Hormone; IHC: Immunohistochemistry; QRT: Querty Real Time.

## Competing interests

The authors all declare that they have no competing interests.

## Authors’ contributions

AB: development of the research plan, material and data collection, performance of immunohistochemical analysis, and manuscript preparation. JB: help in performing immunohistochemical analysis and manuscript preparation. ML: help in research planning and supervision of the immunohistochemical analysis, help in manuscript preparation and correction. Bogdan Rumianowski: manuscript preparation and correction. SS-G: analysis of results and help in writing and correcting the manuscript. IR: help in writing and correcting the manuscript. AS: help in planning and supervision of the study. MZR: manuscript preparation and correction. All authors have read and approved the final manuscript.
